# Supplementary material for: Cortisol levels, heart rate, and autonomic responses in horses during repeated road transport with differently conditioned trucks in a tropical environment
Source: PLoS One. 2024 Sep 6;19(9):e0301885. doi: 10.1371/journal.pone.0301885 (PMC11379227; doi:10.1371/journal.pone.0301885)
Supplement: S4 Fig — ATS; air-conditioned truck with a space load, RR; beat-to-beat interval, HR; heart rate, SDNN; the standard deviation of the normal-to-normal beat interval, RMSSD; Square root of the mean squared differences between successive RR intervals, pNN50; the relative number of the beat-to-beat intervals that differs than 50 ms, TINN; triangular interpolation of normal-to-normal intervals, RRTI; RR triangular index, VLF; very-low-frequency band, LF; low-frequency band, HF; high-frequency band, SD1; the standard deviation of Poincaré plot perpendicular to the line-of-identity, SD2; the standard deviation of Poincaré plot along the line-of-identity, PNS; the parasympathetic nervous system and SNS; the sympathetic nervous system. (DOCX) [file pone.0301885.s004.docx]

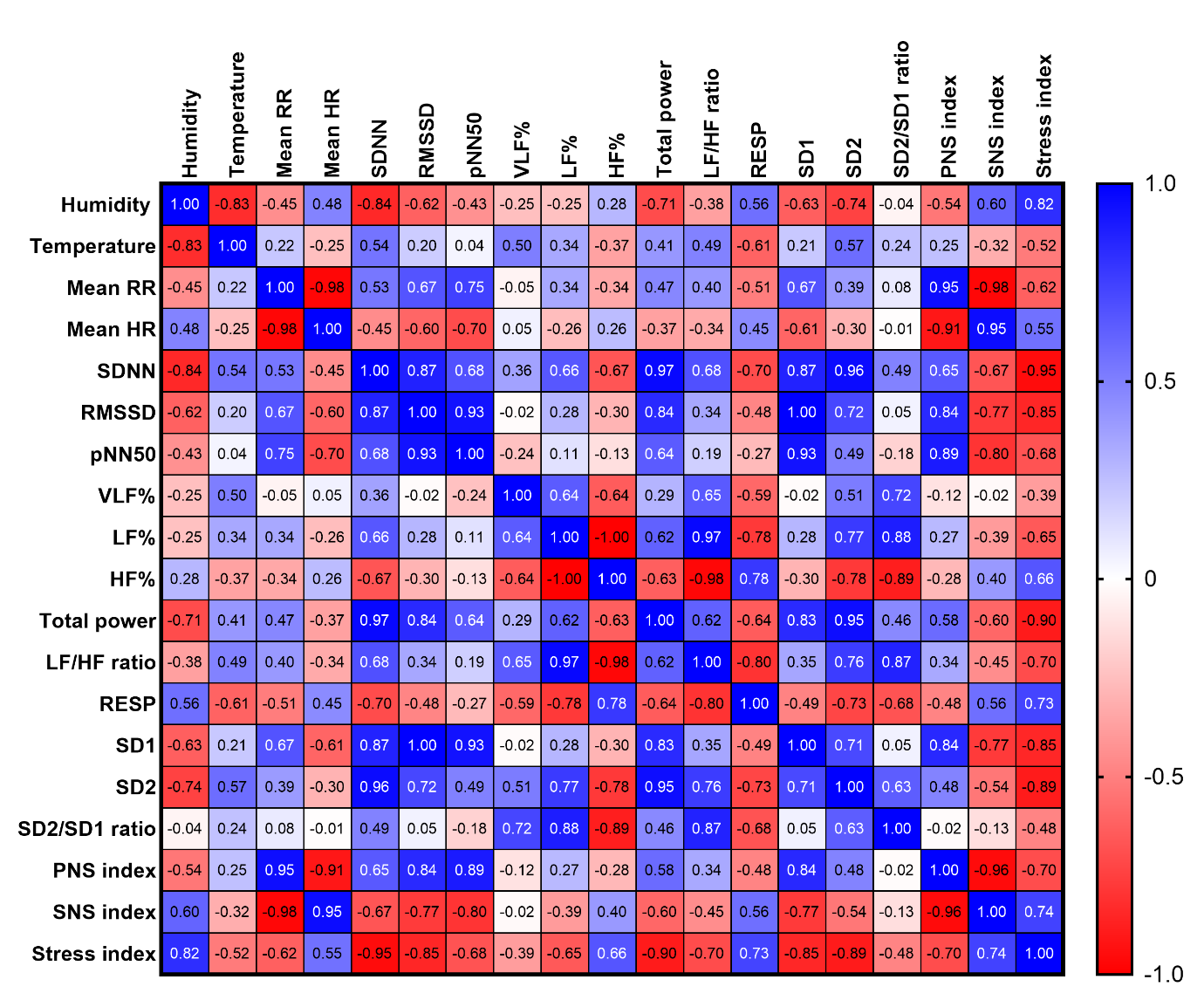


**Fig S4.** Correlation among inside humidity, inside temperature and HRV variables in N-ATS horses during road transport. **N-ATS;** non-air-conditioned truck with a space load, **RR;** beat-to-beat interval, **HR;** heart rate, **SDNN;** the standard deviation of the normal-to-normal beat interval, **RMSSD**; Square root of the mean squared differences between successive RR intervals, **pNN50;** the relative number of the beat-to-beat intervals that differs than 50 ms, **TINN;** triangular interpolation of normal-to-normal intervals, **RRTI**; RR triangular index, **VLF;** very-low-frequency band, **LF;** low-frequency band, **HF;** high-frequency band, **SD1;** the standard deviation of Poincaré plot perpendicular to the line-of-identity, **SD2;** the standard deviation of Poincaré plot along the line-of-identity, **PNS;** the parasympathetic nervous system and **SNS;** the sympathetic nervous system
