# Supplementary material for: Cortisol levels, heart rate, and autonomic responses in horses during repeated road transport with differently conditioned trucks in a tropical environment
Source: PLoS One. 2024 Sep 6;19(9):e0301885. doi: 10.1371/journal.pone.0301885 (PMC11379227; doi:10.1371/journal.pone.0301885)
Supplement: Attachment — Submitted filename: Response to the reviewers.docx [file pone.0301885.s005.docx]

**Response to the editor and reviewers**

***Title*: Cortisol levels, heart rate, and autonomic responses in horses during repeated road transport with differently conditioned trucks in a tropical environment**

Dear Editor and Reviewers,

We’d like to thank you for dedicated time to reviewer our work and give valuable suggestions and comments to improve this manuscript. We’ve addressed all point raised by the editor and reviewers. The corrections are highlighted **in green** while the statements that have already been mentioned are highlighted **in yellow**.

**Journal requirements:**

When submitting your revision, we need you to address these additional requirements.

1. Please ensure that your manuscript meets PLOS ONE's style requirements, including those for file naming. The PLOS ONE style templates can be found at <https://journals.plos.org/plosone/s/file?id=wjVg/PLOSOne_formatting_sample_main_body.pdf> and

<https://journals.plos.org/plosone/s/file?id=ba62/PLOSOne_formatting_sample_title_authors_affiliations.pdf>

**Response to the editor**

We’ve revised the content and headings according to the journal-style templates.

2. Please include your full ethics statement in the ‘Methods’ section of your manuscript file. In your statement, please include the full name of the IRB or ethics committee who approved or waived your study, as well as whether or not you obtained informed written or verbal consent. If consent was waived for your study, please include this information in your statement as well.

**Response to the editor**

We’ve added the verbal consent and ethical approval statement on pages 4-5, lines 88-90.

3. We note that Fig S1 in your submission contain [map/satellite] images which may be copyrighted. All PLOS content is published under the Creative Commons Attribution License (CC BY 4.0), which means that the manuscript, images, and Supporting Information files will be freely available online, and any third party is permitted to access, download, copy, distribute, and use these materials in any way, even commercially, with proper attribution. For these reasons, we cannot publish previously copyrighted maps or satellite images created using proprietary data, such as Google software (Google Maps, Street View, and Earth). For more information, see our copyright guidelines: <http://journals.plos.org/plosone/s/licenses-and-copyright>. We require you to either (1) present written permission from the copyright holder to publish these figures specifically under the CC BY 4.0 license, or (2) remove the figures from your submission:

**Response to the editor**

We decided to remove the S1 Fig from the manuscript to avoid breaching the copyright.

4. Please review your reference list to ensure that it is complete and correct. If you have cited papers that have been retracted, please include the rationale for doing so in the manuscript text, or remove these references and replace them with relevant current references. Any changes to the reference list should be mentioned in the rebuttal letter that accompanies your revised manuscript. If you need to cite a retracted article, indicate the article’s retracted status in the References list and also include a citation and full reference for the retraction notice.
 **Response to the editor**

We’ve modified a bit in the reference list by adding the DOI in all journal article to ease the reader tracking those cited article.

Review Comments to the Author

Please use the space provided to explain your answers to the questions above. You may also include additional comments for the author, including concerns about dual publication, research ethics, or publication ethics. (Please upload your review as an attachment if it exceeds 20,000 characters)

Reviewer #1: This is a very interesting area of study that is important for ensuring best welfare standards in equine transportation management. The manuscript is well written, and uses a comprehensive set of stress response measures nicely, well done. However, it is regrettable that on this occasion the sample size is too small to draw any meaningful conclusions.

I look forward to seeing further research investigating this topic, studied in a larger cohort of horses.

**Response to the reviewer.**

We’d like to thank the reviewer for this kind word. We agree that the sample size is too small, and we have addressed this as the limitation of this study.

Reviewer #2: This was an interesting article. Thorough analysis and well presented. I only had a couple of minor comments

Line 21 use temperate rather than cold climates here

**Response to the reviewer.**

We’ve revised it on **page 2, line 22** accordingly.

Line 22 did you estimate or did you measure these levels / parameters ?

**Response to the reviewer.**

We’ve revised it by using measured instead of estimate on **page 2, line 23** accordingly.

Line 44 number of shows seems low – are you just retrieving data from one discipline – the 150 shows is only 3 per weekend – in Show Jumping alone there are at least 6 to 9 FEI shows each weekend – please check numbers or change text

**Response to the reviewer.**

We’d like to thank the reviewer for this notification. We’ve given wrong information. In fact, there are over 3200 shows organizing annually regarding the FEI database. We’ve revised it on **page 3, line 45** accordingly.

Line 72 temperate rather than cold climates or non-tropical may be better descriptors

Table 1 mean drive speed as assume this speed was not constant throughout trial.

**Response to the reviewer.**

We’ve revised to use the temperate instead of the cold environment on page 4, line 73, accordingly. Regarding the driving speed, the driver has been constructed to drive approximately 90 km/h according to the speed gadget of the truck. However, they can not keep this speed constant throughout the trip as the speed has sometimes been slowed down due to transient traffic jams or waiting to pay a toll fee. The mean driving speed was computed from the polar flow program with respect to the data from one sports watch placed within the truck of each trip.

For materials and methods or similar Given this was travel on a highway is it possible to somehow describe the characteristics of the route ie took 10 mins from loading site until on highway and able to maintain a steady state ? or similar

**Response to the reviewer.**

We agree with the reviewer that describing the characteristics of the route may provide clear information to the reader. However, based on the response to the reviewer above, the driver could not keep the speed of 90 km/h as we instructed throughout the trip due to the transient traffic jam or waiting to pay the toll fee. These factors resulted in different driving speeds between the dates of the experiment, even though the trucks were driven on the same route. So, in our opinion, describing the characteristics of the route may confuse the reader rather than give them useful information. Please correct me if I am wrong.

Reviewer #3: This paper is interesting and provides a good approach to examining the stress responses to trucking conditions. The discussion explains the results with good comparison to other literature

A few minor comments

Please edit p-values so that the p is lowercase and all values are rounded to 3 decimal places.

Table 1 and 3 could do with a bit more information in the title to make it standalone

Were windows closed during transport?

**Response to the reviewer.**

We’ve revised all p-vales in lowercase throughout the text accordingly. In a non-air-conditioned truck, the windows were open throughout the trips.
